# Infections in Patients With Cytokine Release Syndrome/Immune Effector Cell–Associated Neurotoxicity Syndrome Following Chimeric Antigen Receptor T-Cell Therapy

**DOI:** 10.1093/ofid/ofag274

**Published:** 2026-06-10

**Authors:** Antonio Gallardo-Pizarro, Miquel Ariño, Carlos Lopera, Ana Martinez-Urrea, Cynthia Terrones-Campos, Ainhoa Castiella-Aranzasti, E Azucena González-Navarro, Nil Albiol, Christian Teijon-Lumbreras, Cristina Pitart, M Ángeles Marcos, Valentín Ortíz-Maldonado, Josep Mensa, Julio Delgado, Álvaro Urbano-Ispizua, Carlos Fernández de Larrea, Alex Soriano, Nuria Martinez-Cibrian, Pedro Castro, Carolina Garcia-Vidal

**Affiliations:** Infectious Disease Department, Hospital Clinic of Barcelona – Institut d’Investigacions Biomèdiques August Pi i Sunyer (IDIBAPS), Barcelona, Spain; Facultat de Medicina i Ciències de la Salut, Universitat de Barcelona (UB), Barcelona, Spain; Infectious Disease Department, Hospital Clinic of Barcelona – Institut d’Investigacions Biomèdiques August Pi i Sunyer (IDIBAPS), Barcelona, Spain; Infectious Disease Department, Hospital Clinic of Barcelona – Institut d’Investigacions Biomèdiques August Pi i Sunyer (IDIBAPS), Barcelona, Spain; Facultat de Medicina i Ciències de la Salut, Universitat de Barcelona (UB), Barcelona, Spain; Infectious Disease Department, Hospital Clinic of Barcelona – Institut d’Investigacions Biomèdiques August Pi i Sunyer (IDIBAPS), Barcelona, Spain; Facultat de Medicina i Ciències de la Salut, Universitat de Barcelona (UB), Barcelona, Spain; Infectious Disease Department, Hospital Clinic of Barcelona – Institut d’Investigacions Biomèdiques August Pi i Sunyer (IDIBAPS), Barcelona, Spain; Infectious Disease Department, Hospital Clinic of Barcelona – Institut d’Investigacions Biomèdiques August Pi i Sunyer (IDIBAPS), Barcelona, Spain; Department of Immunology, Hospital Clinic of Barcelona, Barcelona, Spain; Hematology Department, Hospital Clinic of Barcelona – Institut d’Investigacions Biomèdiques August Pi i Sunyer (IDIBAPS), Barcelona, Spain; Infectious Disease Department, Hospital Clinic of Barcelona – Institut d’Investigacions Biomèdiques August Pi i Sunyer (IDIBAPS), Barcelona, Spain; Microbiology Department, Hospital Clinic of Barcelona, Institute for Global Health (ISGlobal), Barcelona, Spain; Centro de Investigación Biomédica en Red de Enfermedades Infecciosas (CIBERINFEC), Instituto de Salud Carlos III, Madrid, Spain; Facultat de Medicina i Ciències de la Salut, Universitat de Barcelona (UB), Barcelona, Spain; Microbiology Department, Hospital Clinic of Barcelona, Institute for Global Health (ISGlobal), Barcelona, Spain; Centro de Investigación Biomédica en Red de Enfermedades Infecciosas (CIBERINFEC), Instituto de Salud Carlos III, Madrid, Spain; Hematology Department, Hospital Clinic of Barcelona – Institut d’Investigacions Biomèdiques August Pi i Sunyer (IDIBAPS), Barcelona, Spain; Infectious Disease Department, Hospital Clinic of Barcelona – Institut d’Investigacions Biomèdiques August Pi i Sunyer (IDIBAPS), Barcelona, Spain; Facultat de Medicina i Ciències de la Salut, Universitat de Barcelona (UB), Barcelona, Spain; Hematology Department, Hospital Clinic of Barcelona – Institut d’Investigacions Biomèdiques August Pi i Sunyer (IDIBAPS), Barcelona, Spain; Facultat de Medicina i Ciències de la Salut, Universitat de Barcelona (UB), Barcelona, Spain; Hematology Department, Hospital Clinic of Barcelona – Institut d’Investigacions Biomèdiques August Pi i Sunyer (IDIBAPS), Barcelona, Spain; Facultat de Medicina i Ciències de la Salut, Universitat de Barcelona (UB), Barcelona, Spain; Hematology Department, Hospital Clinic of Barcelona – Institut d’Investigacions Biomèdiques August Pi i Sunyer (IDIBAPS), Barcelona, Spain; Infectious Disease Department, Hospital Clinic of Barcelona – Institut d’Investigacions Biomèdiques August Pi i Sunyer (IDIBAPS), Barcelona, Spain; Facultat de Medicina i Ciències de la Salut, Universitat de Barcelona (UB), Barcelona, Spain; Hematology Department, Hospital Clinic of Barcelona – Institut d’Investigacions Biomèdiques August Pi i Sunyer (IDIBAPS), Barcelona, Spain; Facultat de Medicina i Ciències de la Salut, Universitat de Barcelona (UB), Barcelona, Spain; Medical Intensive Care Unit, Hospital Clinic of Barcelona – Institut d’Investigacions Biomèdiques August Pi i Sunyer (IDIBAPS), Barcelona, Spain; Infectious Disease Department, Hospital Clinic of Barcelona – Institut d’Investigacions Biomèdiques August Pi i Sunyer (IDIBAPS), Barcelona, Spain; Facultat de Medicina i Ciències de la Salut, Universitat de Barcelona (UB), Barcelona, Spain

**Keywords:** CAR T-cell therapy, cytokine release syndrome, immune effector cell–associated neurotoxicity syndrome, infections, multimodal immunosuppression

## Abstract

**Background:**

Cytokine release syndrome (CRS) and immune effector cell–associated neurotoxicity syndrome (ICANS) are common toxicities after chimeric antigen receptor (CAR) T-cell therapy. We evaluated the incidence, timing, microbiology, and risk factors of microbiologically documented infections during the CRS and/or ICANS phase.

**Method:**

Consecutive adults receiving CAR T-cell therapy between 2020 and 2023 were retrospectively analyzed. Infections were classified by type and timing. The 60-day cumulative incidence of first infection and associated factors were assessed using Fine–Gray competing-risk models.

**Results:**

Among 152 CAR T-cell infusions, CRS and/or ICANS occurred in 117 (77.0%) episodes, and 70 (59.8%) required immunomodulatory therapy. An infectious etiology was documented in 30 (25.6%) episodes, yielding 45 documented infections (21 bacterial [46.7%], 17 viral [37.8%], and 7 fungal [15.6%]), more frequent in immunomodulatory-treated episodes (32.9% vs 14.9%; *P* = .033). At infection onset, median temperature, and C-reactive protein (CRP) levels were lower than at CRS/ICANS diagnosis (37.2°C vs 38.4°C; *P* < .001 and 3.9 vs 6.0 mg/L; *P* = .002). In immunomodulatory-treated episodes, median time to first infection was 15 (7–22) days from CRS and 9 (5–20) days from ICANS. The 60-day cumulative infection incidence was 60.0% with multimodal immunosuppression and 20.8% without; multimodal immunosuppression independently increased infection risk (subdistribution hazard ratio [sHR] 3.29; 95% CI 1.38–7.82; *P* = .007).

**Conclusions:**

The CRS/ICANS phase carries a high risk of bacterial, viral, and fungal infections, particularly under multimodal immunosuppression, while fever and CRP are unreliable indicators. Focused strategies for early detection, prevention, and prompt treatment of infections are urgently needed.

## Teaser

Microbiologically documented infections complicate one quarter of cytokine release syndrome (CRS)/immune effector cell–associated neurotoxicity syndrome (ICANS) episodes after chimeric antigen receptor (CAR) T-cell therapy, typically emerging beyond the second week. Multimodal immunosuppression increases infection risk and blunts fever and inflammatory markers, complicating diagnosis and underscoring the need for targeted surveillance strategies.

Chimeric antigen receptor (CAR) T-cell therapy has emerged as a transformative treatment for patients with relapsed or refractory hematological malignancies [1, 2]. However, its use is frequently complicated by 2 interrelated issues, such as immune-mediated toxicities and infectious complications [3–7].

Among immune-related toxicities and their treatments, cytokine release syndrome (CRS), immune effector cell–associated neurotoxicity syndrome (ICANS), and immune effector cell–associated hematotoxicity (ICAHT) are the most prevalent and have been recognized as significant risk factors for subsequent infections [8–12]. Despite this association, data specifically characterizing the clinical and epidemiological features of microbiologically documented infections occurring during episodes of CRS and/or ICANS remain scarce.

A clearer understanding of infection dynamics during this vulnerable period is critical to improve prevention and diagnostic strategies and tailor antimicrobial management. This study characterizes microbiologically documented infections occurring during CRS and/or ICANS in patients receiving CAR T-cell therapy for hematological malignancies, incorporating stratification by immunomodulatory treatment. We also evaluated clinical and laboratory findings at CRS/ICANS diagnosis and at infection onset to help characterize these 2 events, which are often difficult to distinguish clinically, and we estimated the 60-day cumulative incidence of documented infection and associated risk factors.

## METHODS

### Study Design and Setting

This study was conducted at the Hospital Clinic of Barcelona (HCB), whose Hematology Department covers a referral area of more than 1.5 million inhabitants, while its CAR T-cell program operates as a national referral center. Hospital Clinic of Barcelona has fully developed 2 academic CAR T-cell constructs: varnimcabtagene autoleucel (ARI-0001 or var-cel), an anti-CD19 product evaluated in the multicenter CART19-BE-01 trial for relapsed/refractory CD19-positive malignancies, and cesnicabtagene autoleucel (ARI0002h or cesni-cel), a humanized B-cell maturation antigen (BCMA)-directed CAR T-cell investigated in the CARTBCMA-HCB-01 pilot study for relapsed/refractory multiple myeloma [13–15]. Both products are approved by the Spanish Medicines Agency (AEMPS) under the Hospital Exemption clause, which allows patients nationwide to be treated with these products at HCB.

According to institutional practice, immune-mediated toxicities were monitored daily from the day of CAR T-cell administration until day +28 and were managed according to institutional protocols based on recommendations from the European Society for Blood and Marrow Transplantation (EBMT) and the Joint Accreditation Committee of International Society for Cell & Gene Therapy (ISCT) and EBMT (JACIE) and the European Hematology Association (EHA) [16]. Infections were recorded through day +60 after CAR T-cell infusion. Antibacterial prophylaxis with levofloxacin (500 mg once daily) was administered during periods of absolute neutrophil count (ANC) ≤ 500 cells/mm^3^ and discontinued upon neutrophil recovery. Antifungal prophylaxis with fluconazole (400 mg once daily) was initiated at day 0 and continued through day +30. Mold-active prophylaxis with isavuconazole (200 mg once daily after an initial loading dose) or posaconazole (300 mg once daily after an initial loading dose) was prescribed at the treating physician's discretion, although it was strongly recommended in patients receiving immunomodulatory therapy for CRS/ICANS, those with prolonged neutropenia (>14 days), or those with prior invasive mold infection. When used, it was continued for at least 1 month after corticosteroid discontinuation. Trimethoprim–sulfamethoxazole (160/800 mg) was given 3 times weekly from lymphodepletion until at least 18 months after infusion, CD4 count > 250 cells/mm^3^, and CD19 recovery. Antiviral prophylaxis with acyclovir (800 mg once or twice daily) or valacyclovir (500 mg twice daily) was administered from the start of lymphodepletion until at least 18 months after infusion, once herpes zoster vaccination was completed. Cytomegalovirus (CMV) DNAemia was monitored by serial plasma polymerase chain reaction (PCR) in high-risk patients according to institutional practice, including those with preinfusion CMV DNAemia, persistent lymphocytopenia < 200 cells/mm^3^, grade ≥ 3 cytokine release syndrome (CRS), receipt of ≥2 immunosuppressive agents, or corticosteroid exposure for >3 days, with weekly monitoring in those requiring immunomodulatory therapy for CAR T-cell–related toxicities until 30 days after corticosteroid withdrawal. Antiviral therapy was initiated pre-emptively—generally at CMV DNAemia > 1000 IU/mL—or therapeutically, according to viral load, kinetics, clinical presentation, and physician judgment. Serum immunoglobulin G (IgG) levels were measured prior to infusion and monthly thereafter; intravenous immunoglobulin replacement (400 mg/kg) was indicated in patients with IgG levels < 400 mg/dL and who had experienced at least 1 infectious episode. Postinfusion fever or suspected infection was managed per institutional protocol, as previously described [17].

### Selection of Participants

Between January 2020 and March 2023, all consecutive adult patients who received CAR T-cell therapy at our institution, either hospital-based products (ie, var-cel and cesni-cel) or commercially available products (axicabtagene ciloleucel or tisagenlecleucel), were eligible for inclusion. Varnimcabtagene autoleucel and cesni-cel were administered in escalating fractionated schedules, containing 10%, 30%, and 60% of the target dose, depending on whether serious immune-related events occurred. Accordingly, fractionated infusion schedules were considered a single treatment episode. Cesnicabtagene autoleucel additionally had a preplanned booster dose administered ≥100 days after the initial infusion in responders, which was also considered part of the same episode. Episodes were included irrespective of whether the planned infusion schedule was fully completed.

### Data Collection

Data were retrieved from the electronic health records of all eligible patients. Collected variables included demographic information, underlying hematological malignancy, baseline clinical status, and the type of CAR T-cell products administered. The occurrence, timing, and severity of CRS and/or ICANS were recorded, along with specific treatments used for their management. For each microbiologically documented infection, we collected epidemiological data, site of infection, causative pathogens, antimicrobial susceptibility profiles, and clinical outcomes. Vital signs and laboratory parameters at the time of microbiological sampling—including ANC, absolute lymphocyte count, and C-reactive protein (CRP) concentration—were systematically recorded.

### Definitions

Infectious episodes were defined according to Centers for Disease Control and Prevention/National Healthcare Safety Network criteria as microbiologically confirmed infections accompanied by compatible clinical manifestations; fungal infections were classified according to the revised European Organization for Research and Treatment of Cancer/Mycosis Study Group consensus definitions [18, 19]. Infection onset was defined as the date of collection of the microbiological sample establishing the diagnosis of infection. All episodes were reviewed by the authors (A. G.- P., M. A., and C. G.- V.).

Cytokine release syndrome and ICANS were assessed and graded according to the American Society for Transplantation and Cellular Therapy (ASTCT) consensus criteria [20]. For the descriptive analysis, immunomodulatory therapy for CRS/ICANS was defined as the administration of any systemic corticosteroid course (excluding hydrocortisone), interleukin-6 receptor antagonist (tocilizumab), interleukin-6 antagonist (siltuximab), or interleukin-1 receptor antagonist (anakinra) during the toxicity episode. The timing and cumulative doses of tocilizumab were documented, and administration of anakinra and siltuximab was recorded. All systemic corticosteroid courses were registered, with corticosteroid duration defined as the total number of days with systemic exposure, and high-dose corticosteroid therapy (ie, dexamethasone 10 mg every 6 hours for ≥1 day or methylprednisolone 500 mg/day for ≥3 days). An additional composite variable, referred to as multimodal immunosuppression, was defined to identify CRS/ICANS episodes in which both multiple tocilizumab administrations (≥2 doses) and a high-dose corticosteroid course occurred.

### Statistical Analysis

The primary objective was to describe the occurrence and clinical characteristics of all microbiologically documented infections during CRS and/or ICANS episodes. Secondary objectives included comparing clinical and laboratory parameters at CRS/ICANS onset versus infection onset, evaluating infection risk in relation to immunomodulatory therapy, estimating the 60-day cumulative incidence of documented infection, and identifying variables associated with infection.

Continuous variables were reported as medians with interquartile ranges (IQRs) and categorical variables as counts and percentages. Comparisons between groups were performed using the Mann–Whitney U test for continuous data and Fisher's exact or χ^2^ tests for categorical data, as appropriate.

The first microbiologically documented infection was analyzed as a time-dependent variable within 60 days after CAR T-cell infusion using Fine–Gray models, with death considered a competing event. Exploratory univariable analyses were first performed to identify variables associated with infection. Categorical variables with a prevalence of <10% and variables with >30% missing values were excluded. Continuous variables were standardized per SD. A multivariable model was then used to adjust computed subdistribution hazard ratios (sHRs) and 95% CIs. A 2-sided *P* < .05 was considered statistically significant.

Infection density was calculated among episodes receiving multimodal immunosuppression as the number of infection events per patient-days at risk, multiplied by 100. All analyses were conducted using R software, version 4.5.1 (R Foundation for Statistical Computing, Vienna, Austria), and Dataiku Data Science Studio, version 14.0.2 (Dataiku, Paris, France).

### Ethics

The study protocol was approved by the institutional ethics committee (HCB/2025/0936), with a waiver of informed consent granted in accordance with institutional policy and the principles of the Declaration of Helsinki. Reporting follows the Strengthening the Reporting of Observational Studies in Epidemiology (STROBE) guidelines [21].

## RESULTS

### Cohort Characteristics and Immunomodulatory Therapies

**Figure 1.**
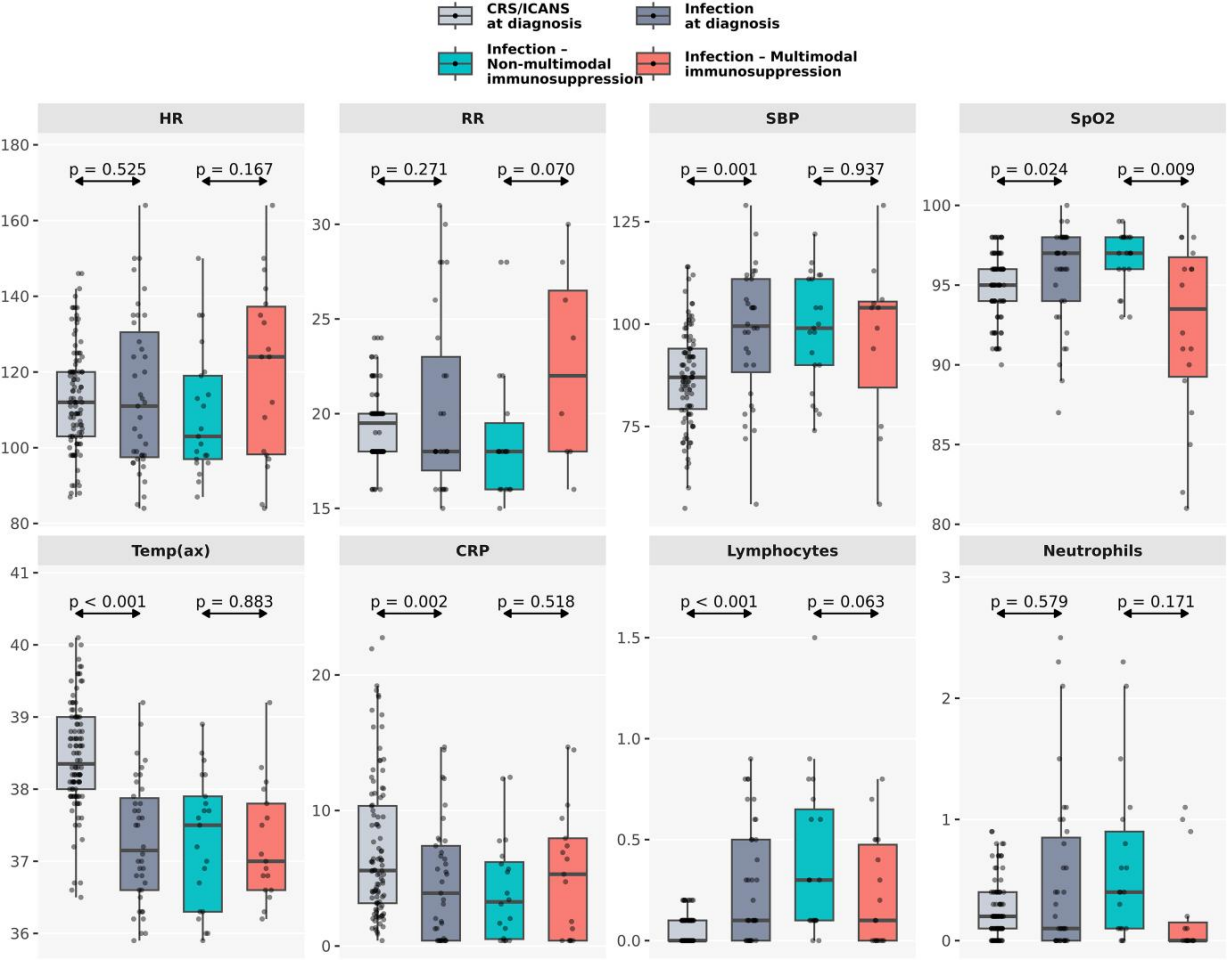
Vital signs and laboratory parameters at cytokine release syndrome (CRS)/immune effector cell–associated neurotoxicity syndrome (ICANS) onset and at infection diagnosis, with infection cases stratified by multimodal immunosuppression. Infectious episodes were stratified according to receipt of multimodal immunomodulatory therapy (≥2 doses of tocilizumab plus high-dose corticosteroids vs none). Vital signs and laboratory parameters were recorded at CRS/ICANS onset and at the time of infection diagnosis. Vital signs: heart rate (HR; beats/min), respiratory rate (RR; breaths/min), systolic blood pressure (SBP; mmHg), temperature (axillary) (Temp(ax); °C), and peripheral oxygen saturation (SpO_2_; %). Laboratory parameters: C-reactive protein (CRP; mg/L), lymphocyte count (×10^9^/L), and neutrophil count (×10^9^/L). Outliers were excluded from visualization using the 1.5× interquartile range (IQR) rule; all data were included in statistical testing (Wilcoxon rank-sum test).

During the study period, 141 patients with hematological malignancies received 152 CAR T-cell infusions. Baseline characteristics are shown in Table 1. Any-grade CRS occurred in 117 of 152 infusions (77.0%), and 70 of 117 episodes (59.8%) required immunomodulatory therapy. Immune effector cell–associated neurotoxicity syndrome developed after 18 of 152 infusions (11.8%). At CRS/ICANS diagnosis, the median temperature was 38.4°C (IQR, 38.0–39.0) and the median CRP level was 6.0 mg/L (IQR, 3.2–11.2) (Figure 1). At CRS/ICANS diagnosis, 508 microbiological samples were collected across 102 episodes, of which only 11 (2.2%) were positive (Supplementary Table 1). Cytokine release syndrome/ICANS severity and corresponding immunomodulatory treatments, including timing, are summarized in Table 2. A study flow diagram is provided in Supplementary Figure 1.

**Table 1.** Demographics and Baseline Characteristics

Variable	N = 152
Age (years), median (IQR)	54 (37–64)
Females, n (%)	85 (55.9)
**Underlying hematological disease, n (%)**	
Acute lymphoblastic leukemia	54 (35.5)
Multiple myeloma	30 (19.7)
Non-Hodgkin lymphoma	61 (40.1)
Diffuse large B-cell lymphoma	35 (23.0)
Follicular lymphoma	9 (5.9)
Mantle cell lymphoma	5 (3.3)
Primary mediastinal B-cell lymphoma	4 (2.6)
Burkitt lymphoma	2 (1.3)
High-grade B-cell lymphoma	2 (1.3)
Nodal marginal zone lymphoma	1 (0.7)
Primary CNS lymphoma	1 (0.7)
Small B-cell lymphoma	1 (0.7)
Transformed follicular lymphoma	1 (0.7)
Richter's transformation from chronic lymphocytic leukemia	7 (4.6)
Prior HSCT, n (%)	83 (54.6)
Allogeneic HSCT	51 (33.6)
Autologous HSCT	32 (21.1)
Time from prior HSCT, months, median (IQR)	…
Allogeneic	15 (8.5–30.0)
Autologous	30 (8.0–58.9)
**CAR T-cell product received, n (%)**	
Varnimcabtagene autoleucel (ARI-0001)	87 (57.2)
Axicabtagene ciloleucel	30 (19.7)
Cesnicabtagene autoleucel (ARI0002h)	29 (19.1)
Other CAR T-cell products	6 (3.9)
**Comorbidities, n (%)**	
Hypertension	24 (15.8)
Chronic heart disease	10 (6.6)
Chronic liver disease	9 (5.9)
Previous solid tumor	8 (5.3)
Chronic kidney disease	7 (4.6)
Chronic pulmonary disease	6 (3.9)
Diabetes mellitus	5 (3.3)
**Pre-CAR-T laboratory values, median** (**IQR**)	
Serum IgG levels (mg/dL)^a^	520 (320–620)
Absolute lymphocyte count (×10^9^/L)	0.1 (0–0.1)
Absolute neutrophil count (×10^9^/L)	0.7 (0.3–1.2)

Abbreviations: CAR T-cell, chimeric antigen receptor T-cell; CNS, central nervous system; HSCT, hematopoietic stem cell transplant; IgG, immunoglobulin G.

^**a**^Serum IgG levels were available for 27 of 152 episodes.

**Table 2.** Characteristics of Toxicity and Immunomodulatory Therapy in CRS and/or ICANS

Variables	N = 70
**CRS (per ASTCT criteria), n (%)**	
Grade 1	38 (54.3)
Grade 2	27 (38.6)
Grade 3	5 (7.1)
Grade 4	0 (0.0)
**ICANS (per ASTCT criteria), n (%)**	
Grade 1	6 (8.6)
Grade 2	7 (10.0)
Grade 3	3 (4.3)
Grade 4	2 (2.9)
**Immunomodulatory therapy, n (%)**	
Receipt of tocilizumab	65 (92.9)
1 dose	38 (54.3)
2 doses	11 (15.7)
≥3 doses	16 (22.9)
Receipt of anakinra	7 (10.0)
Receipt of siltuximab	2 (2.9)
Receipt of corticosteroids	39 (55.7)
High-dose corticosteroid therapy, n (%)	24 (34.3)
Duration (days), median (IQR)	13 (5–18)
**Days between timepoints, median (IQR)**	
From infusion to toxicity onset	4 (1–9)
From infusion to tocilizumab administration	8 (4–11)
From infusion to anakinra administration	18 (8–20)
From infusion to corticosteroid initiation	8 (6–13)
From infusion to first documented infection	22 (11–28)
From CRS to first documented infection	15 (7–22)
From ICANS to first documented infection	9 (5–20)
From tocilizumab to first documented infection	14 (5–19)
From corticosteroids to first documented infection	10 (2–24)

Percentages in subheadings refer to the number of individuals who developed the corresponding toxicity and received the corresponding therapeutic intervention, not to the entire cohort. Duration of corticosteroids refers to the number of days with any systemic corticosteroid exposure (excluding hydrocortisone). *High-dose corticosteroid therapy* is defined as dexamethasone 10 mg every 6 hours for ≥1 day or methylprednisolone 500 mg/day for ≥3 days.

Abbreviations: ASTCT, American Society for Transplantation and Cellular Therapy; CRS, cytokine release syndrome; ICANS, immune effector cell–associated neurotoxicity syndrome.

### Epidemiology of Infections

**Figure 2.**
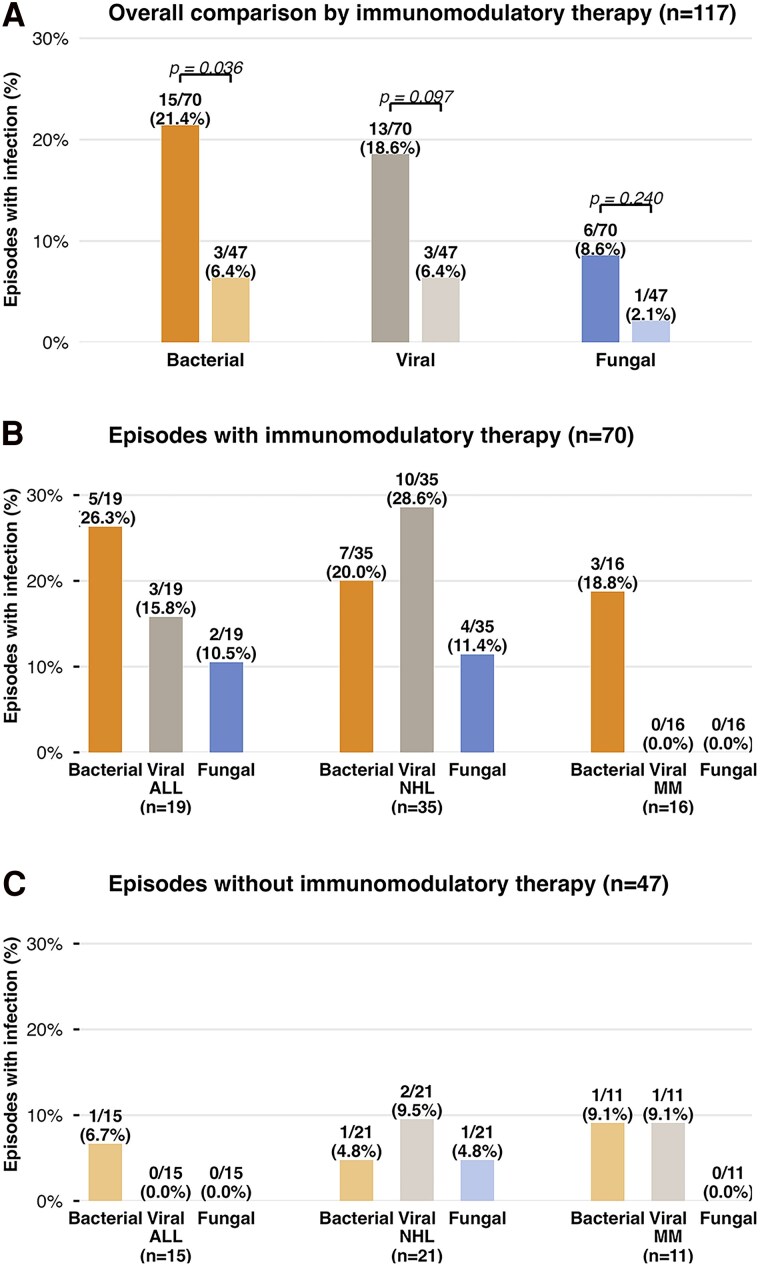
Bacterial, viral, and fungal infections according to immunomodulatory therapy and underlying hematological disease in chimeric antigen receptor (CAR) T-cell therapy episodes with cytokine release syndrome (CRS) and/or immune effector cell–associated neurotoxicity syndrome (ICANS). (*A*) Comparison of the proportion of episodes with bacterial, viral, and fungal infections between episodes with and without immunomodulatory therapy (darker vs lighter bars, respectively). (*B* and *C*) The distribution of infections stratified by underlying hematological disease among episodes with immunomodulatory therapy (*B*) and without immunomodulatory therapy (*C*). *P*-values were calculated using Fisher's exact test. Abbreviations: ALL, acute lymphoblastic leukemia; MM, multiple myeloma; NHL, non-Hodgkin lymphoma.

During CRS/ICANS, at least 1 infection was documented in 30/117 (25.6%) episodes. Overall, these 30 episodes accounted for 45 infections: 21/45 (46.7%) bacterial, 17/45 (37.8%) viral, and 7/45 (15.6%) fungal. The median time from CRS onset to the first infection was 14 days (IQR, 6–20). Detailed characteristics of bacterial infections, including antecedent beta-lactam exposure and breakthrough episodes, are provided in Supplementary Table 2. Cytomegalovirus was documented in 7/117 (6.0%) CRS/ICANS episodes and accounted for 7/17 (41.2%) viral infections. Detailed characteristics, management, and outcomes of CMV episodes are provided in Supplementary Table 3. Fungal infections occurred in 6/117 (5.1%) CRS/ICANS episodes, comprising 7 fungal events overall (3 yeast and 4 mold); 6/7 occurred during azole prophylaxis (Supplementary Table 4). All infections are summarized in Supplementary Table 5. Infection rates according to immunomodulatory therapy and underlying hematological disease, expressed per episode at risk, are shown in Figure 2.

**Figure 3.**
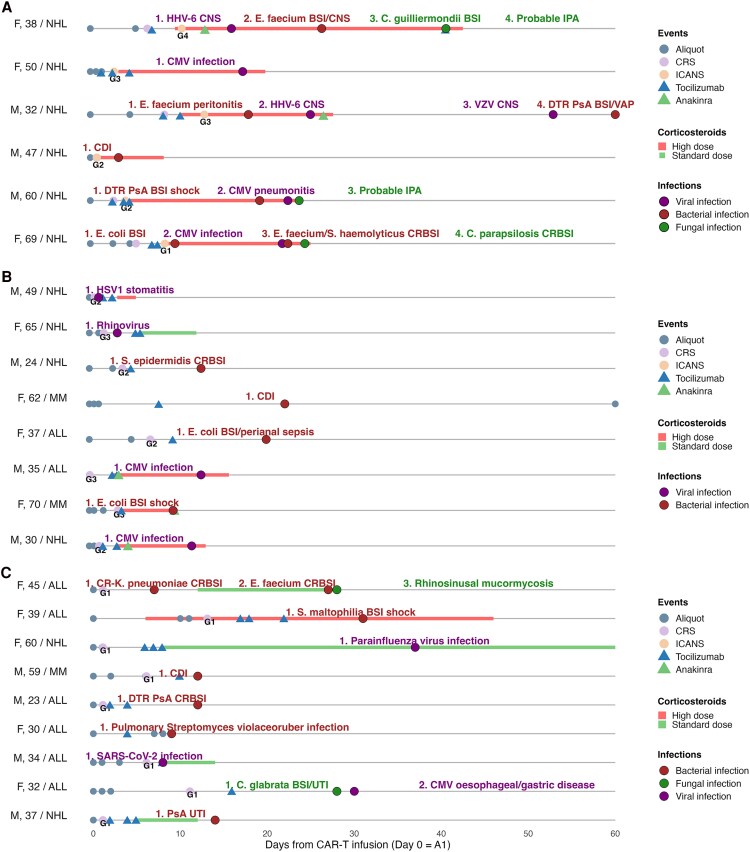
Infections and clinical events in patients with CRS and/or ICANS requiring immunomodulatory therapy after CAR-T infusion: (*A*) CRS with concomitant ICANS (n = 18), (*B*) CRS grades 2–4 without ICANS (n = 22), and (*C*) CRS grade 1 without ICANS (n = 30). Each horizontal lane represents 1 patient (sex, age, and hematologic disease on the left). Time is anchored at day 0 (first infusion, *A*1). Circles indicate aliquot collections (*A*1–*A*3; in cesnicabtagene autoleucel, *A*4 is omitted). Upward triangles denote administration of tocilizumab or anakinra . Horizontal bars indicate systemic corticosteroids (high dose or standard dose). Circles indicate infections categorized by etiology (bacterial, viral, or fungal) and annotated above the timeline. Abbreviations: ALL, acute lymphoblastic leukemia; BSI, bloodstream infection; CDI, *Clostridioides difficile* infection; CMV, cytomegalovirus; CNS, central nervous system; CR, carbapenem-resistant; CRBSI, catheter-related bloodstream infection; CRS, cytokine release syndrome; DTR, difficult-to-treat; HHV-6, human herpesvirus 6; HSV-1, herpes simplex virus 1; ICANS, immune effector cell–associated neurotoxicity syndrome; IPA, invasive pulmonary aspergillosis; MM, multiple myeloma; NHL, non-Hodgkin lymphoma; PsA, *Pseudomonas aeruginosa*; SARS-CoV-2, severe acute respiratory syndrome coronavirus 2; TCZ, tocilizumab; UTI, urinary tract infection; VAP, ventilator-associated pneumonia; VZV, varicella-zoster virus.

A total of 23/70 (32.9%) CRS/ICANS episodes with immunomodulatory therapy versus 7/47 (14.9%) without immunomodulatory therapy were complicated by infections (*P* = .033). Among the 23 episodes with infections in patients receiving immunomodulatory therapy, 1 infection was observed in 17/23 (73.9%), 2 in 1/23 (4.3%), and ≥3 in 5/23 (21.7%). The median time from CRS onset to the first infection in this subgroup was 15 days (IQR, 7–22). At least 1 bacterial infection occurred in 15/23 (65.2%) episodes, at least 1 viral infection in 12/23 (52.2%), and at least 1 fungal infection in 5/23 (21.7%). Overall, these 23 episodes accounted for 37 microbiologically documented infectious events: 18/37 (48.6%) bacterial, 13/37 (35.1%) viral, and 6/37 (16.2%) fungal. In CRS/ICANS episodes managed with immunomodulatory therapy, Figure 3 shows the distribution of therapies and associated infections, stratified into CRS with concomitant ICANS (*A*), CRS grades 2–4 (*B*), and CRS grade 1 (*C*).

Documented infections in CRS/ICANS episodes not requiring immunomodulatory therapy are described in Supplementary Table 6, with bacterial infections reported in 3/7 (42.9%) episodes, viral infections in 3/7 (42.9%), and fungal infections in 1/7 (14.3%). In these episodes, the median time from CRS onset to the first infection was 13 days (IQR, 2–18).

**Figure 4.**
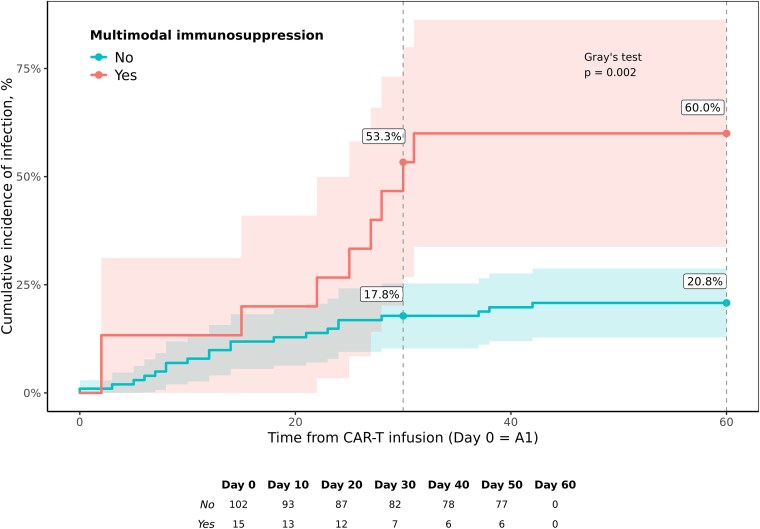
Cumulative incidence of the first microbiologically documented infection by multimodal immunosuppression status. Numbers at risk represent patients remaining under observation at each time point, stratified according to multimodal immunosuppression status. Follow-up was administratively censored at day 60. Abbreviations: A1, aliquot 1; CAR-T, chimeric antigen receptor T-cell.

At 30 and 60 days after CAR T-cell infusion, the cumulative incidence of the first infection was 53.3% and 60.0%, respectively, among patients receiving multimodal immunosuppression, compared with 17.8% and 20.8% among those who did not (Figure 4). The infection density within 60 days was higher in CRS plus ICANS than in CRS only for any infection (2.80 vs 1.17 per 100 patient-days at risk among patients receiving multimodal immunosuppression (Supplementary Figure 2).

### Clinical and Laboratory Findings at Infection Onset

At infection onset, the median temperature was 37.2°C (IQR, 36.6–37.9) compared with 38.4°C (IQR, 38.0–39.0) at CRS/ICANS diagnosis (*P* < .001). Among patients receiving multimodal immunomodulatory therapy, the median temperature was 37.0°C (IQR, 36.6–37.8) versus 37.5°C (IQR, 36.3–37.9) in those not treated (*P* = .883). The median CRP levels were 3.9 mg/L (IQR, 0.4–7.4) at infection onset versus 6.0 mg/L (IQR, 3.2–11.2) at CRS/ICANS diagnosis (*P* = .002). In addition, the median CRP levels were 5.3 mg/L (IQR, 0.4–8.0) in patients receiving multimodal therapy versus 3.3 mg/L (IQR, 0.5–6.2) in those not treated (*P* = .518). Figure 1 summarizes vital signs and laboratory parameters at CRS/ICANS diagnosis and at infection onset stratified by immunomodulatory therapy.

### Risk Factors for Infections

**Figure 5.**
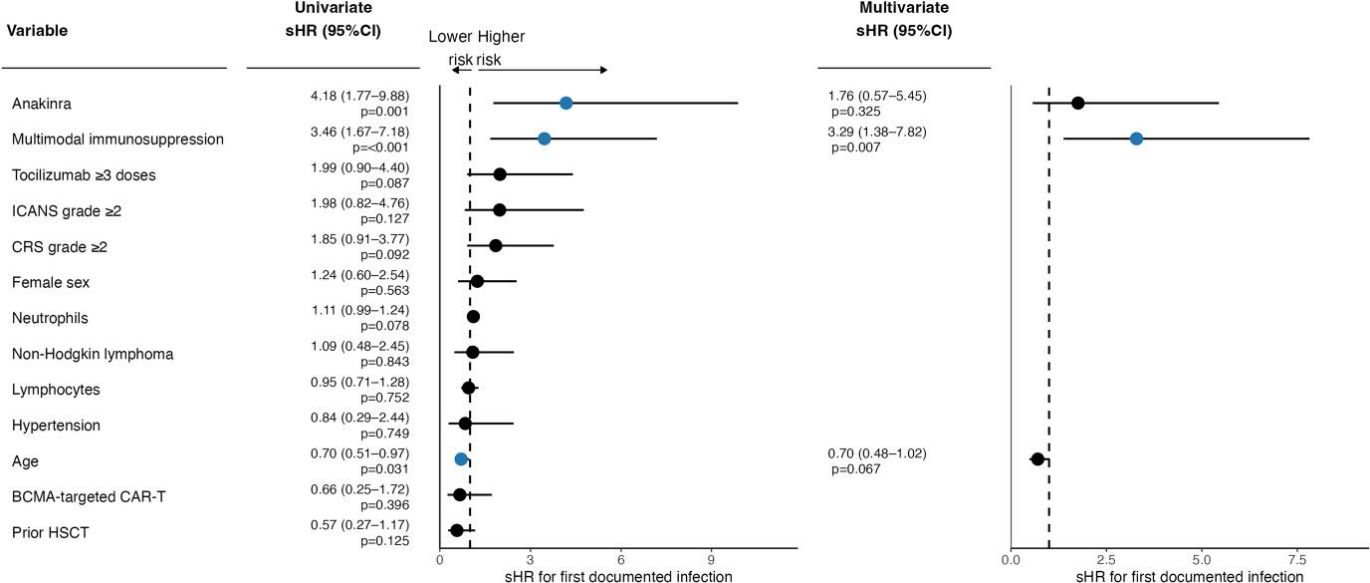
Forest plot of univariable and multivariable Fine–Gray competing-risk analyses for factors associated with the first infection, with death as a competing event. Subdistribution hazard ratios (sHRs), 95% CIs, and *P-*values are presented. Abbreviations: BCMA, B-cell maturation antigen; CAR-T, chimeric antigen receptor T-cell; CRS, cytokine release syndrome; HSCT, hematopoietic stem cell transplant; ICANS, immune effector cell–associated neurotoxicity syndrome; sHR, subdistribution hazard ratio.

Anakinra and multimodal immunosuppression administered for CRS or ICANS were associated with a higher risk of microbiologically documented infection (sHR = 4.18; 95% CI, 1.77–9.88; *P* = .001 and sHR = 3.46; 95% CI, 1.67–7.18; *P* < .001, respectively), whereas age (per 1-SD increase) was associated with a lower risk (sHR_per1SD_ = 0.70; 95% CI, 0.51–0.97; *P* = .031) (Figure 5) in univariate analysis. In a multivariable model, only multimodal immunosuppression was associated with a higher risk of first infection (sHR = 3.29; 95% CI, 1.38–7.82; *P* = .007).

### Mortality

The overall all-cause mortality was 16 of 141 patients (11.3%). Mortality was 20.0% (6/30) in episodes with infection versus 8.2% (10/122) in episodes without infection (*P* = .09). Among the six deaths in the infection group, 4 were adjudicated as infection attributable. The median time from the first documented infection to death in these cases was 19 days (IQR, 6.5–34).

## DISCUSSION

In this retrospective study, approximately 1 in 4 CRS and/or ICANS episodes in patients with hematological malignancies receiving CAR T-cell therapy was complicated by a microbiologically documented infection. Although most patients showed clinical signs suggestive of infection, such as fever, tachycardia, or hypotension, microbiological confirmation at CRS/ICANS onset was uncommon, consistent with previous reports [17, 22]. Infections in our cohort generally occurred later, usually beyond 2 weeks after CRS/ICANS diagnosis.

Understanding this temporal shift in infection risk is critical for prevention and diagnostic strategies. Infections arising during established CRS or ICANS, particularly in patients treated with immunomodulatory therapy, were frequently not accompanied by conventional clinical markers, such as fever or elevated CRP, reflecting the immunosuppressive effects of corticosteroids and IL-6 inhibitors. This phenomenon, previously described as “cold sepsis” [23], poses a significant diagnostic challenge. Clinicians should recognize that early febrile neutropenia is often noninfectious, whereas later infections may present subtly, often with signs of physiological compromise despite a blunted febrile response. This underscores the need for vigilant monitoring and a high index of suspicion.

Previous studies have suggested that mucosal barrier disruption and the systemic inflammation associated with immune-mediated toxicities contribute to infection risk. However, our data highlight a marked difference in infection incidence between patients who did and did not receive immunomodulatory therapy, with episodes managed without such therapy showing a markedly lower risk. Most infections occurred in the setting of profound neutropenia, with immunosuppressive treatments, including high-dose corticosteroids or tocilizumab for CRS and ICANS, representing key predisposing factors [10, 24]. In this context, the inverse association between age and infection risk observed in univariate analysis may reflect confounding by indication, related to age-dependent differences in the CAR T-cell products administered and the higher CRS burden reported in younger patients, with consequent greater exposure to immunomodulatory therapies [3].

The majority of infections in our population were of bacterial origin, consistent with previous reports [4, 10, 24–26]. However, viral infections were also common, occurring in half of infected patients, with CMV reactivation representing a frequent event among patients exposed to high-dose corticosteroids. This likely reflects the profound and sustained lymphodepletion driven by the underlying hematological malignancy and prior treatments, immunosuppressive therapy for CAR T-cell immune–related toxicities, ICAHT, and CAR T-cell therapy itself. Remarkably, fungal infections occurred in 9% of CRS/ICANS episodes receiving immunomodulatory therapy, exceeding the approximately 3% incidence reported in large CAR T-cell cohorts, and underscoring the heightened risk in this subgroup [27–29].

The optimal strategy for infection prevention and early diagnosis in patients with CRS/ICANS complicating CAR T-cell therapy has yet to be established [4, 26, 30]. Consistent with our findings, we suggest that following CRS/ICANS diagnosis, particularly in patients receiving immunosuppressive therapy, future studies should evaluate the potential benefit of routine blood cultures every 48 hours, even in afebrile patients, alongside serial CMV PCR testing and fungal surveillance (eg, galactomannan or PCR), especially in those not receiving mold-active prophylaxis. However, interpretation may be limited by false-positive or clinically insignificant findings and should be evaluated in prospective studies [27].

This study has several limitations, including its retrospective design, the heterogeneity in CAR T-cell products and diseases treated, and the evolution of uniform, standardized protocols for infectious screening across the cohort. Our study focused on infections during CRS/ICANS episodes; delayed toxicities, such as non-ICANS neurotoxicity associated with BCMA-targeting products, which may also require immunomodulatory therapy and contribute to infection risk, were not evaluated. Likewise, immune effector cell–associated hemophagocytic lymphohistiocytosis-like syndrome was not systematically assessed, as diagnostic criteria were not yet established during the study period. The small number of infection-related deaths limited the statistical power to determine the prognostic impact of infections in this setting. Nonetheless, these data provide real-world evidence of the infectious burden during CRS and ICANS and identify temporal and clinical features that should alert to and guide clinicians in the management of infections in this high-risk population.

In conclusion, microbiologically documented bacterial, viral, and fungal infections were frequently observed during the CRS/ICANS phase after CAR T-cell therapy. Fever and inflammatory markers are poor indicators due to blunting by multimodal immunosuppression used for toxicity management. Defining effective strategies for prevention, early diagnosis, and treatment of these infections should be a research priority.

## Supplementary Material

ofag274_Supplementary_Data

## Supplementary Data

Supplementary materials are available at *Open Forum Infectious Diseases* online. Consisting of data provided by the authors to benefit the reader, the posted materials are not copyedited and are the sole responsibility of the authors, so questions or comments should be addressed to the corresponding author.
