# Supplementary material for: Infections in Patients With Cytokine Release Syndrome/Immune Effector Cell–Associated Neurotoxicity Syndrome Following Chimeric Antigen Receptor T-Cell Therapy
Source: Open Forum Infect Dis. 2026 Jun 10;13(6):ofag274. doi: 10.1093/ofid/ofag274 (PMC13251341; doi:10.1093/ofid/ofag274)
Supplement: ofag274_Supplementary_Data [file ofag274_supplementary_data.docx]

**Online Supplement to “Infections in patients with CRS/ICANS following CAR T-cell therapy”**

Antonio Gallardo-Pizarro, Miquel Ariño, Carlos Lopera, Ana Martinez-Urrea, Cynthia Terrones-Campo, Ainhoa Castiella-Aranzasti, E Azucena González-Navarro, Nil Albiol, Christian Teijon-Lumbreras, Cristina Pitart, M. Ángeles Marcos, Valentín Ortíz-Maldonado, Josep Mensa, Julio Delgado, Álvaro Urbano-Ispizua, Carlos Fernández de Larrea, Alex Soriano, Nuria Martinez-Cibrian, Pedro Castro, Carolina Garcia-Vidal

**Contents**

**Supplementary Table S1.** Microbiological results from samples obtained within 48 hours of CRS/ICANS diagnosis.

**Supplementary Table S2.** Characteristics of bacterial infections during CRS/ICANS.

**Supplementary Table S3.** Characteristics of CMV episodes during CRS/ICANS.

**Supplementary Table S4.** Characteristics of fungal infections during CRS/ICANS.

**Supplementary Table S5.** Documented infections in patients with CRS and/or ICANS.

**Supplementary Table S6.** Timeline from CRS/ICANS onset to first documented infection in patients not receiving immunomodulatory therapy.

**Supplementary Figure S1.** Flowchart of patient selection, CAR T-cell infusions, and treatment of CAR T-cell–related toxicities.

**Supplementary Figure S2.** Infection density within 60 days from CRS and/or ICANS onset among episodes receiving multimodal immunosuppression, stratified as CRS only versus CRS plus ICANS.

**Supplementary Table S1. Microbiological results from samples obtained within 48 hours of CRS/ICANS diagnosis.**

| Sample type | Episodes with samples (n) | Total samples (n) | Positive samples (n) | Microorganisms identified |
| --- | --- | --- | --- | --- |
| Blood culture | 100 | 233 | 4 | *Escherichia coli*, *Rothia mucilaginosa*, *Staphylococcus haemolyticus*, *Roseomonas mucosa* |
| Urine culture | 93 | 107 | 0 | — |
| Respiratory sample^†^ | 46 | 62 | 4 | SARS-CoV-2 (n = 2), Adenovirus, Enterovirus |
| Stool sample^‡^ | 22 | 24 | 1 | *Clostridioides difficile* |
| Other^§^ | 54 | 82 | 2 | CMV, HSV-1 |

^†^ Respiratory samples were mainly nasopharyngeal swabs analyzed by multiplex respiratory virus PCR panels; one bronchoalveolar lavage and one sputum sample were also included.

^‡^ Stool samples included multiplex enteropathogenic PCR panels, conventional stool cultures, and *Clostridioides difficile* toxin assays.

^§^ “Other” includes 48 episodes (56 samples) analyzed by fungal antigen assays (galactomannan and β-D-glucan), 17 episodes (20 samples) tested by targeted viral PCR assays in whole blood (HSV-1/2, HHV-6/8, CMV, EBV, VZV, Parvovirus B19, Enterovirus, *Toxoplasma gondii*), 5 episodes (5 samples) with cerebrospinal fluid analyses (multiplex meningoencephalitis PCR, JC virus PCR, lymphocytic choriomeningitis virus PCR, antigen assays for *Cryptococcus neoformans* and *Streptococcus pneumoniae,* and culture), and 1 skin biopsy sample.

*Abbreviations*: **CRS**, cytokine release syndrome; **ICANS**, immune effector cell–associated neurotoxicity syndrome; **PCR**, polymerase chain reaction; **HSV**, herpes simplex virus; **HHV**, human herpesvirus; **CMV**, cytomegalovirus; **EBV**, Epstein–Barr virus; **VZV**, varicella–zoster virus.

**Supplementary Table S2. Characteristics of bacterial infections during CRS/ICANS.**

| **Patient (sex, age / underlying disease)** | **CRS grade** | **ICANS grade** | **Immunomodulatory therapy*** | **Empirical antibiotics during CRS/ICANS^†^** | **Antibiotic prophylaxis** | **Breakthrough^‡^** | **Antibiotics at infection diagnosis^§^** | | | **Infection** | | **Outcome** |
| --- | --- | --- | --- | --- | --- | --- | --- | --- | --- | --- | --- | --- |
| F, 38 / NHL | G2 | G4 | Methylprednisolone 1 g/day ×12 days; tocilizumab ×3; siltuximab ×4; anakinra ×46 days | Meropenem 1 g q8h ×3 days + amikacin 15 mg/kg q24h ×2 days → ceftazidime–avibactam 2.5 g q8h + daptomycin 10 mg/kg q24h + metronidazole 500 mg q8h ×10 days → piperacillin–tazobactam 4.5 g q8h ×5 days | Levofloxacin 500 mg q24h | No | Tigecycline 100 mg q12h + daptomycin 10 mg/kg q24h → vancomycin 1 g q8h (including intrathecal) + linezolid 600 mg q8h | | | Bacteremic meningitis (*E. faecium*) | | Infection-related mortality |
| M, 32 / NHL | G2 | G3 | Dexamethasone 20 mg q8h ×8days; tocilizumab ×2; anakinra ×10 days | Piperacillin–tazobactam 4.5 g q8h ×5 days → meropenem 1 g q8h ×7 days + amikacin 20 mg/kg q24h x4 days + Teicoplanin 400 mg q12h x 4 days | Levofloxacin 500 mg q24h | No | Piperacillin–tazobactam 4.5 g q8h + teicoplanin 400 mg q12h | | | Secondary peritonitis due to gastric perforation (*E. faecium*) | | Non–infection-related mortality (disease progression) |
|  |  |  |  |  | No | Yes | Ceftazidime–avibactam 2.5 g q8h + nebulised amikacin | | | Bacteremic VAP (DTR *P. aeruginosa*) | |  |
| M, 47 / NHL | G1 | G2 | Dexamethasone 10 mg q6h ×6days | Meropenem 1 g q8h ×12 days | No | No | Vancomycin 125 mg q6h | | | *Clostridioides difficile* infection | | Clinical resolution |
| F, 60 / NHL | G1 | G2 | Dexamethasone 10 mg q6h ×10days; tocilizumab ×3 | Piperacillin–tazobactam 4.5 g q8h ×2days → meropenem 1 g q8h ×2 days → piperacillin–tazobactam 4.5 g q8h ×2days | Levofloxacin 500 mg q24h | No | Ceftazidime–avibactam 2.5 g q8h → ceftolozane/tazobactam 1.5 g q8h + amikacin 15 mg/kg q24h | | | Bacteremic septic shock (DTR *P. aeruginosa*) | | Infection-related mortality |
| F, 69 / NHL | G3 | G1 | Dexamethasone 10 mg q6h ×6 days; Tocilizumab ×3 | Piperacillin–tazobactam 4.5 g q8h ×7days → meropenem 1 g q8h ×2 days | Levofloxacin 500 mg q24h | No | | Meropenem 2 g q8h + amikacin 15 mg/kg q24h | | | Bacteremic septic shock (*E. coli*) | Infection-related mortality |
|  |  |  |  |  | No | Yes | | Piperacillin–tazobactam 4.5 g q12h + daptomycin 8 mg/kg q48h | | | CRBSI (*E. faecium* + *S. haemolyitcus*) |  |
| M, 24 / NHL | G2 | — | Tocilizumab ×1 | Meropenem 1 g q8h ×2 days → piperacillin–tazobactam 4.5 g q8h ×10days | Levofloxacin 500 mg q24h | No | | Meropenem 1 g q8h + daptomycin 10 mg/kg q24h | | | CRBSI (*S. epidermidis*) | Clinical resolution |
| F, 62 / MM | G2 | — | Dexamethasone 20 mg q24h ×1 day; Tocilizumab ×1 | Meropenem 1 g q8h ×6 days → ceftazidime–avibactam 2.5 g q8h ×2 days | Levofloxacin 500 mg q24h | No | | Metronidazole 500 mg q8h | | | *Clostridioides difficile* infection | Clinical resolution |
| F, 37 / ALL | G2 | — | Tocilizumab ×1 | Meropenem 1 g q8h ×10 days + teicoplanin 400 mg q12h ×3 days → piperacillin–tazobactam 4.5 g q8h ×3days → meropenem 1 g q8h ×8 days + amikacin 20 mg/kg q24h ×1 days + teicoplanin 400 mg q12h ×2 days | Levofloxacin 500 mg q24h | No | | Meropenem 1 g q8h + amikacin 20 mg/kg q24h + daptomycin 10 mg/kg q24h | | | Perianal-source bacteremia (3GCephRE *E. coli*) | Clinical resolution |
| F, 70 / MM | G3 | — | Methylprednisolone 1 g q24h ×1 day; dexamethasone 10 mg q6h ×5 days; tocilizumab ×1; anakinra ×1 day | Meropenem 1 g q8h ×5 days + teicoplanin 400 mg q12h ×2 days | Levofloxacin 500 mg q24h | No | | | Meropenem 1 g q8h + amikacin 15 mg/kg q24h + teicoplanin 400 mg q12h | | Septic shock with bacteremia (3GCephRE *E. coli*) | Infection-related mortality |
| F, 45 / ALL | G1 | — | Dexamethasone 10 mg q8h ×3 days | Meropenem 1 g q8h ×6 days | Levofloxacin 500 mg q24h | No | | | Ceftazidime–avibactam 2.5 g q8h + amikacin 25 mg/kg q24h | | CRBSI (*K. pneumoniae* ESBL+OXA-48 | Clinical resolution |
|  |  |  |  |  | No | Yes | | | Ceftazidime–avibactam 2.5 g q8h + daptomycin 10 mg/kg q24h | | CRBSI (*E. faecium*) |  |
| F, 39 / ALL | G1 | — | Dexamethasone 10 mg q6h ×5 days; tocilizumab ×3 | Piperacillin–tazobactam 4.5 g q8h ×5 days → meropenem 1 g q8h ×9 days + teicoplanin 400 mg q12h ×8 days | No | Yes | | | Ceftazidime–avibactam 2.5 g q8h + tigecycline 100 mg q12h + co-trimoxazole 1600 mg q6h + levofloxacin 500 mg q12h | | Septic shock with bacteremia (*S. maltophilia*) | Clinical resolution |
| M, 59 / MM | G1 | — | Tocilizumab ×1 | Meropenem 1 g q8h ×8 days + teicoplanin 400 mg q12h ×2 days | Levofloxacin 500 mg q24h | No | | | Vancomycin 125 mg q6h | | *Clostridioides difficile* infection | Clinical resolution |
| M, 23 / ALL | G1 | — | Tocilizumab ×2 | Meropenem 1 g q8h ×6 days | Levofloxacin 500 mg q24h | No | | | Ceftolozane/tazobactam 1.5 g q8h | | CRBSI (DTR *P. aeruginosa*) | Clinical resolution |
| F, 30 / ALL | G1 | — | Tocilizumab ×1 | Piperacillin–tazobactam 4.5 g q8h ×10 days + amikacin 15 mg/kg q24h ×3 days + vancomycin 1 g q8h ×10 days | No | Yes | | | Meropenem 1 g q8h | | Pulmonary infection (*Streptomyces violaceoruber*) | Clinical resolution |
| M, 37 / NHL | G1 | — | Tocilizumab ×4 | Meropenem 1 g q8h ×4 days → piperacillin–tazobactam 4.5 g q8h ×4days | Levofloxacin 500 mg q24h | No | | | Piperacillin–tazobactam 4.5 g q8h | | UTI (*P. aeruginosa*) | Clinical resolution |
| M, 55 / MM | G1 | — | — | Meropenem 1 g q8h ×1 day | Levofloxacin 500 mg q24h | No | | | Meropenem 1 g q8h | | CRBSI (*Roseomonas mucosa)* | Clinical resolution |
| F, 38 / NHL | G1 | — | — | Meropenem 1 g q8h ×4 days | No | No | | | Vancomycin 125 mg q6h | | *Clostridioides difficile* infection | Clinical resolution |
| F, 43 / ALL | G1 | — | — | Meropenem 1 g q8h ×5 days → piperacillin–tazobactam 4.5 g q8h ×3days | Levofloxacin 500 mg q24h | No | | | Meropenem 1 g q8h + teicoplanin 400 mg q12h | | CRBSI (*E. coli* + *E. faecium*) | Clinical resolution |

* For corticosteroids, duration (days) corresponds to the number of days at the maximum recorded dose during each CRS/ICANS episode.

^†^ Empirical antibiotics during CRS/ICANS were defined as all systemic antibacterial agents administered from CRS/ICANS onset until discontinuation or transition to prophylactic therapy.

^‡^ Breakthrough infection was defined as a microbiologically documented bacterial infection occurring during active systemic antibacterial therapy, irrespective of in vitro susceptibility of the isolated pathogen; infections occurring during fluoroquinolone prophylaxis alone were not considered breakthrough.

^§^ Antibiotics at infection diagnosis were defined as the antibacterial regimen administered within 24 hours of microbiological sample collection establishing the diagnosis of infection.

*Abbreviations*: **3GCephRE**, third-generation cephalosporin-resistant Enterobacterales; **ALL**, acute lymphoblastic leukaemia; **CRBSI**, catheter-related bloodstream infection; **CRS**, cytokine release syndrome; **DTR**, difficult-to-treat resistance; **ICANS**, immune effector cell–associated neurotoxicity syndrome; **MM**, multiple myeloma; **NHL**, non-Hodgkin lymphoma; **UTI**, urinary tract infection; **VAP**, ventilator-associated pneumonia.

**Supplementary Table S3.** **Characteristics of CMV episodes during CRS/ICANS.**

| **Patient (sex, age / underlying disease)** | **CRS grade** | **ICANS grade** | **Immunomodulatory therapy*** | **Peak CMV viral load (IU/mL)** | **Clinical presentation** | **Antiviral therapy** | **Outcome** |
| --- | --- | --- | --- | --- | --- | --- | --- |
| F, 50 / NHL | G3 | G3 | Methylprednisolone 1 g/day ×3 days; tocilizumab ×3 | 8,310 | CMV DNAemia treated pre-emptively | Foscarnet | Virological clearance |
| F, 69 / NHL | G3 | G1 | Dexamethasone 10 mg q6h ×6days; tocilizumab ×3 | 514 | CMV DNAemia | None | Infection-related mortality (non–CMV-related) |
| M, 35 / ALL | G3 | — | Dexamethasone 20 mg q6h ×7days; tocilizumab ×3; anakinra ×14 days | 16,800 | CMV DNAemia treated pre-emptively | Foscarnet | Virological clearance |
| M, 30 / NHL | G2 | — | Dexamethasone 10 mg q6h ×10 days; tocilizumab ×2; anakinra ×7 days | 2,210 | CMV DNAemia treated pre-emptively | Ganciclovir | Virological clearance |
| F, 60 / NHL | G1 | G2 | Dexamethasone 10 mg q6h ×10days; tocilizumab ×3 | Plasma 10,200; BAL 45,900 | CMV pneumonitis | Foscarnet | Infection-related mortality |
| F, 32 / ALL | G1 | — | Tocilizumab ×1 | 3,091 | CMV oesophageal/gastric disease | Ganciclovir | Clinical resolution |
| F, 56 / MM | G1 | — | — | 1,120 | CMV DNAemia | None | Virological clearance |

* For corticosteroids, duration (days) corresponds to the number of days at the maximum recorded dose during each CRS/ICANS episode.

*Abbreviations*: **BAL**, bronchoalveolar lavage; **CMV**, cytomegalovirus; **CRS**, cytokine release syndrome; **ICANS**, immune effector cell–associated neurotoxicity syndrome.

**Supplementary Table S4.** **Characteristics of fungal infections during CRS/ICANS.**

| **Patient (sex, age / underlying disease)** | **CRS grade** | **ICANS grade** | **Immunomodulatory therapy*** | **Fungal infection** | **EORTC/MSG classification** | **Diagnostic criteria** | **Antifungal prophylaxis** | **Antifungal therapy** | **Outcome** |
| --- | --- | --- | --- | --- | --- | --- | --- | --- | --- |
| F, 38 / NHL | G2 | G4 | Methylprednisolone 1 g/day ×12 days; tocilizumab ×3; siltuximab ×4; anakinra ×46 days | Invasive pulmonary aspergillosis | Probable | Serum GM+; compatible chest CT | Isavuconazole | Isavuconazole + anidulafungin | Infection-related mortality |
|  |  |  |  | *Candida guilliermondii* BSI | Proven | Blood cultures+ | Isavuconazole | Anidulafungin |  |
| F, 69 / NHL | G3 | G1 | Dexamethasone 10 mg q6h ×6days; tocilizumab ×3 | *Candida parapsilosis* CRBSI | Proven | Blood cultures+ | Fluconazole | Anidulafungin | Infection-related mortality |
| F, 60 / NHL | G1 | G2 | Dexamethasone 10 mg q6h ×10days; tocilizumab ×3 | Invasive pulmonary aspergillosis | Probable | BAL GM+; compatible chest CT | Isavuconazole | Isavuconazole + anidulafungin + nebulized amphotericin B | Infection-related mortality |
| F, 45 / ALL | G1 | — | Dexamethasone 10 mg q8h ×3 days | Rhino-sinusal mucormycosis | Proven | Culture *Syncephalastrum racemosum*; compatible histology | Isavuconazole | Posaconazole + amphotericin B IV + topical + surgery | Clinical resolution |
| M, 32 / ALL | G1 | — | Tocilizumab ×1 | *Candida glabrata* BSI (urinary source) | Proven | Blood cultures+; urine culture+ | — | Anidulafungin | Clinical resolution |
| F, 67 / NHL | G1 | — | — | Invasive pulmonary aspergillosis | Probable | BAL culture A*. fumigatus*; compatible chest CT | Fluconazole | Posaconazole | Non–infection-related mortality (disease progression) |

* For corticosteroids, duration (days) corresponds to the number of days at the maximum recorded dose during each CRS/ICANS episode.

*Abbreviations*: **BAL**, bronchoalveolar lavage; **BSI**, bloodstream infection; **CRBSI**, catheter-related bloodstream infection; **CRS**, cytokine release syndrome; **ICANS**, immune effector cell–associated neurotoxicity syndrome; **GM**, galactomannan; **CT**, computed tomography; **EORTC/MSG**, European Organization for Research and Treatment of Cancer/Mycoses Study Group.

**Supplementary Table S5. Documented infections in patients with CRS and/or ICANS.**

| Patient  (sex, age / underlying disease) | CRS grade | ICANS grade | Immunomodulatory therapy | Infection |
| --- | --- | --- | --- | --- |
| F, 45 / ALL | G1 |  | Steroids | CR-*Klebsiella pneumoniae* CRBSI |
|  |  |  |  | *Enterococcus faecium* CRBSI |
|  |  |  |  | Rhinosinusal mucormycosis |
| F, 39 / ALL | G1 |  | Tocilizumab, steroids | *Stenotrophomonas maltophilia* BSI shock |
| F, 60 / NHL | G1 |  | Tocilizumab, steroids | Parainfluenza virus infection |
| M, 59 / MM | G1 |  | Tocilizumab | *Clostridioides difficile* infection |
| M, 23 / ALL | G1 |  | Tocilizumab | DTR-*Pseudomonas aeruginosa* CRBSI |
| F, 30 / ALL | G1 |  | Tocilizumab | Pulmonary *Streptomyces violaceoruber* infection |
| M, 34 / ALL | G1 |  | Tocilizumab, steroids | SARS-CoV-2 infection |
| F, 32 / ALL | G1 |  | Tocilizumab | *Candida glabrata* BSI/UTI |
|  |  |  |  | CMV esophageal/gastric disease |
| M, 37 / NHL | G1 |  | Tocilizumab, steroids | *Pseudomonas aeruginosa* UTI |
| F, 38 / NHL | G1 |  | None | *Clostridioides difficile* infection |
| F, 43 / ALL | G1 |  | None | *Escherichia coli* and *Enterococcus faecium* CRBSI |
| F, 68 / NHL | G1 |  | None | SARS-CoV-2 infection |
| M, 55 / MM | G1 |  | None | *Roseomonas mucosa* CRBSI |
| F, 56 / MM | G1 |  | None | CMV infection |
|  |  |  |  | SARS-CoV-2 infection |
| M, 49 / NHL | G1 |  | None | Respiratory adenovirus infection |
| F, 67 / NHL | G1 |  | None | Probable invasive pulmonary aspergillosis |
| M, 47 / NHL | G1 | G2 | Steroids | *Clostridioides difficile* infection |
| M, 60 / NHL | G1 | G2 | Tocilizumab, steroids | DTR-*Pseudomonas aeruginosa* BSI shock |
|  |  |  |  | CMV pneumonitis |
|  |  |  |  | Probable IPA |
| M, 49 / NHL | G2 |  | Tocilizumab, steroids | HSV-1 stomatitis |
| M, 24 / NHL | G2 |  | Tocilizumab | *Staphylococcus epidermidis* CRBSI |
| F, 62 / MM | G2 |  | Tocilizumab | *Clostridioides difficile* infection |
| F, 37 / ALL | G2 |  | Tocilizumab | *Escherichia coli* BSI/perianal sepsis |
| M, 30 / NHL | G2 |  | Tocilizumab, anakinra, steroids | CMV infection |
| M, 32 / NHL | G2 | G3 | Tocilizumab, anakinra, steroids | *Enterococcus faecium* peritonitis |
|  |  |  |  | HHV-6 CNS infection |
|  |  |  |  | VZV CNS infection |
|  |  |  |  | DTR-*Pseudomonas aeruginosa* BSI/VAP |
| F, 38 / NHL | G2 | G4 | Tocilizumab, anakinra, siltuximab, steroids | HHV-6 CNS infection |
|  |  |  |  | *Enterococcus faecium* BSI/CNS infection |
|  |  |  |  | *Candida guillermondii* BSI |
|  |  |  |  | Probable IPA |
| F, 65 / NHL | G3 |  | Tocilizumab, steroids | Rhinovirus infection |
| M, 35 / ALL | G3 |  | Tocilizumab, anakinra, steroids | CMV infection |
| F, 70 / MM | G3 |  | Tocilizumab, anakinra, siltuximab, steroids | *Escherichia coli* BSI shock |
| F, 69 / NHL | G3 | G1 | Tocilizumab, steroids | *Escherichia coli* BSI |
|  |  |  |  | CMV infection |
|  |  |  |  | *Enterococcus faecium*/ *Staphylococcus haemolyticus* CRBSI |
|  |  |  |  | *Candida parapsilosis* CRBSI |
| F, 50 / NHL | G3 | G3 | Tocilizumab, steroids | CMV infection |

*Abbreviations*: **ALL**, acute lymphoblastic leukemia; **BSI**, bloodstream infection; **CMV**, cytomegalovirus; **CNS**, central nervous system; **CR**, carbapenem-resistant; **CRBSI**, catheter–related bloodstream infection; **CRS**, cytokine release syndrome; **DTR**, difficult-to-treat resistance; **G,** grade; **HHV-6**, human herpesvirus 6; **HSV-1**, herpes simplex virus type 1; **ICANS**, immune effector cell–associated neurotoxicity syndrome; **IPA**, invasive pulmonary aspergillosis; **MM**, multiple myeloma; **NHL**, non-Hodgkin lymphoma; **SARS-CoV-2**, severe acute respiratory syndrome coronavirus 2; **UTI**, urinary tract infection; **VAP**, ventilator-associated pneumonia; **VZV**, varicella-zoster virus.

**Supplementary Table S6. Timeline from CRS/ICANS onset to first documented infection in patients not receiving immunomodulatory therapy.**

| Patient (sex, age / underlying disease) | CRS grade | ICANS grade | First documented infection | Days from CRS/ICANS onset to first documented infection |
| --- | --- | --- | --- | --- |
| F, 67 / NHL | G1 |  | Probable invasive pulmonary aspergillosis | 16 |
| F, 38 / NHL | G1 |  | *Clostridioides difficile* infection | 21 |
| F, 43 / ALL | G1 |  | *Escherichia coli* and *Enterococcus faecium* CRBSI | 13 |
| F, 68 / NHL | G1 |  | SARS-CoV-2 infection | 20 |
| M, 55 / MM | G1 |  | *Roseomonas mucosa* CRBSI | 1 |
| F, 56 / MM* | G1 |  | CMV infection | 3 |
| M, 49 / NHL | G1 |  | Respiratory adenovirus infection | 1 |

*This patient developed a second infection by SARS-CoV-2 at day 11 from CRS onset.

*Abbreviations*: **ALL**, acute lymphoblastic leukemia; **CMV**, cytomegalovirus; **CRBSI**, catheter–related bloodstream infection; **CRS**, cytokine release syndrome; **ICANS**, immune effector cell–associated neurotoxicity syndrome; **MM**, multiple myeloma; **NHL**, non-Hodgkin lymphoma.

**Supplementary Figure S1. Flowchart of patient selection, CAR T-cell infusions, and treatment of CAR T-cell–related toxicities.**

**
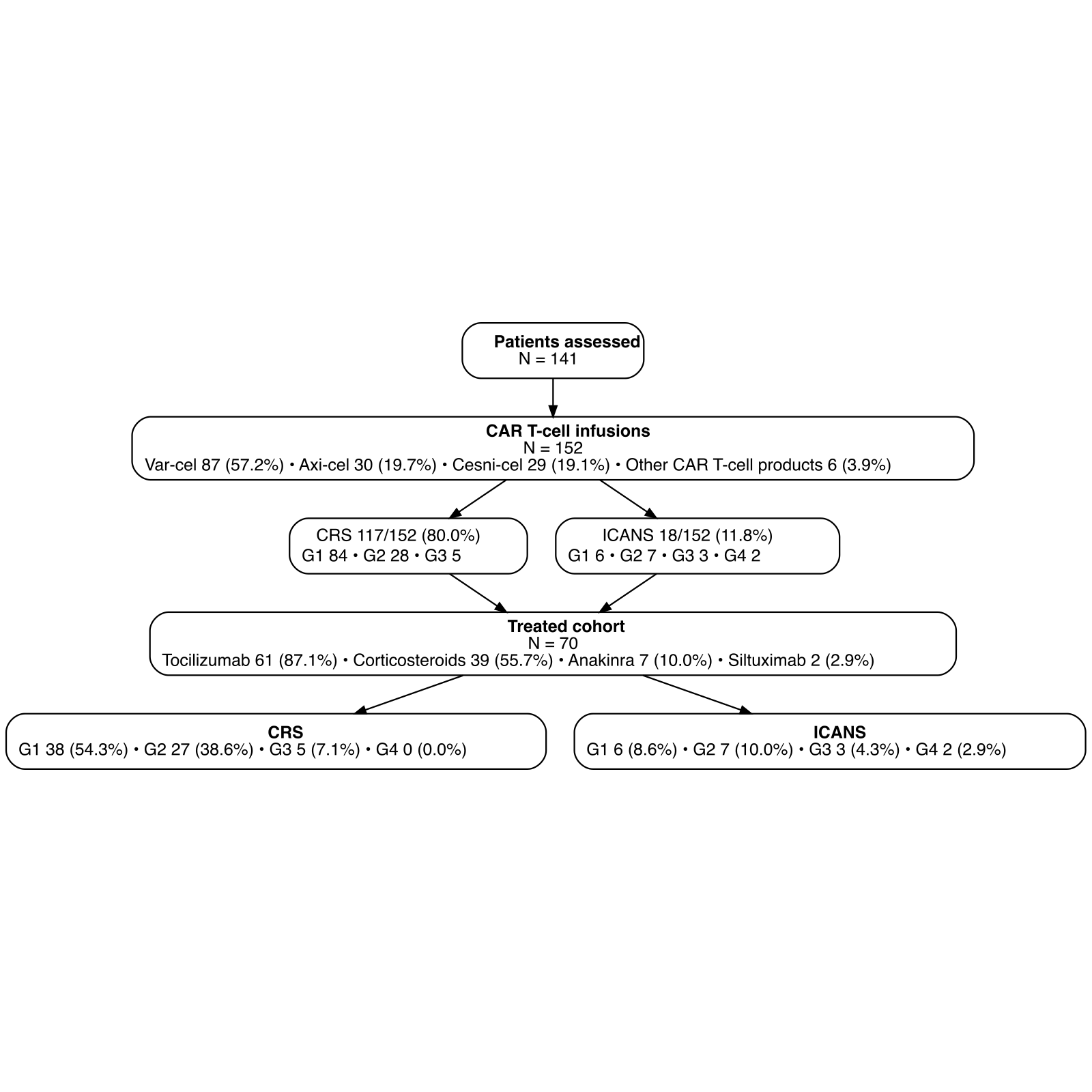
**

Flowchart depicting the selection of 141 patients who underwent 152 CAR T-cell infusion episodes. The incidence and severity of cytokine release syndrome (CRS) and immune effector cell-associated neurotoxicity syndrome (ICANS) are reported according to ASTCT criteria. The treated cohort (n = 70) comprises patients receiving ≥1 targeted intervention (tocilizumab, corticosteroids, anakinra, or siltuximab).

**Supplementary Figure S2. Infection density within 60 days from CRS and/or ICANS onset among episodes receiving multimodal immunosuppression, stratified as CRS only versus CRS plus ICANS.**

**
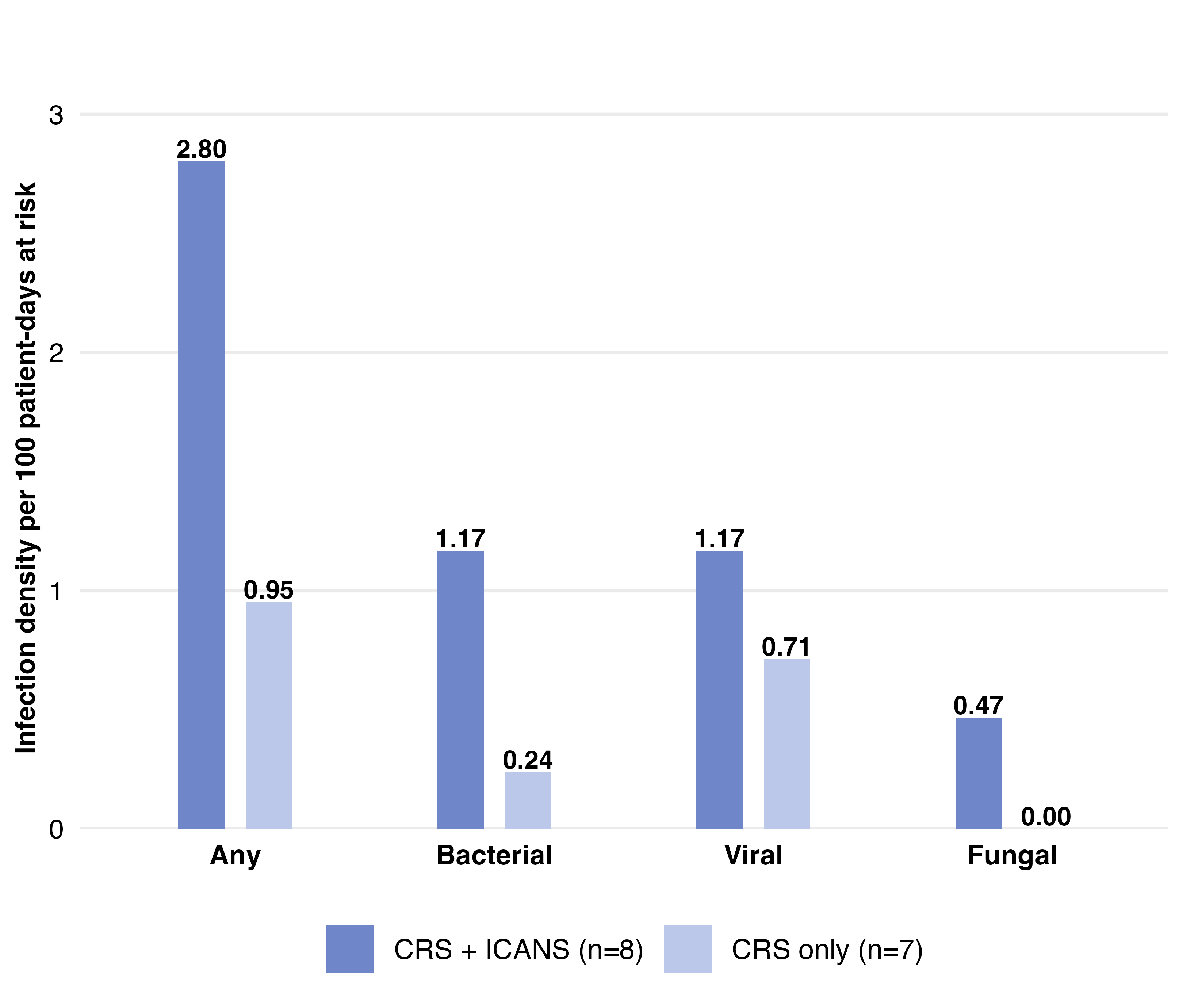
**

Infection density was calculated as the total number of infection events per patient-days at risk multiplied by 100. Only time accrued during person-days at risk contributed to these estimates. Infection densities are shown for any infection and for bacterial, viral, and fungal infections.

*Abbreviations*: **CRS**, cytokine release syndrome; **ICANS**, immune effector cell–associated neurotoxicity syndrome.
